# Production of Sophorolipid Biosurfactant by Insect Derived Novel Yeast *Metschnikowia churdharensis* f.a., sp. nov., and Its Antifungal Activity Against Plant and Human Pathogens

**DOI:** 10.3389/fmicb.2021.678668

**Published:** 2021-06-04

**Authors:** Alka Kumari, Sumeeta Kumari, G. S. Prasad, Anil Kumar Pinnaka

**Affiliations:** Microbial Type Culture Collection and Gene Bank (MTCC), CSIR-Institute of Microbial Technology, Chandigarh, India

**Keywords:** *Fusarium*, antifungal, sophorolipid, biosurfactant, *Metschnikowia*

## Abstract

Biosurfactants are potential biomolecules that have extensive utilization in cosmetics, medicines, bioremediation and processed foods. Yeast produced biosurfactants offer thermal resistance, antioxidant activity, and no risk of pathogenicity, illustrating their promising use in food formulations. The present study is aimed to assess potential of biosurfactant screened from a novel yeast and their inhibition against food spoilage fungi. A novel asexual ascomycetes yeast strain CIG-6A^T^ producing biosurfactant, was isolated from the gut of stingless bee from Churdhar, HP, India. The phylogenetic analysis revealed that the strain CIG-6A^T^ was closely related to *Metschnikowia koreensis*, showing 94.38% sequence similarity in the D1D2 region for which the name *Metschnikowia churdharensis* f.a., sp. nov., is proposed. The strain CIG-6A^T^ was able to produce sophorolipid biosurfactant under optimum conditions. Sophorolipid biosurfactant from strain CIG-6A^T^ effectively reduced the surface tension from 72.8 to 35 mN/m. Sophorolipid biosurfactant was characterized using TLC, FTIR, GC-MS and LC-MS techniques and was a mixture of both acidic and lactonic forms. Sophorolipid assessed promising activity against pathogenic fungi viz. *Fusarium oxysporum* (MTCC 9913), *Fusarium solani* (MTCC 350), and *Colletotrichum gloeosporioides* (MTCC 2190). The inhibitory effect of biosurfactant CIG-6A^T^ against *F. solani* was studied and MIC was 49 μgm/ml, further confirmed through confocal laser scanning microscopy. We illustrated the antifungal activity of sophorolipid biosurfactant from *Metschnikowia* genus for the first time and suggested a novel antifungal compound against food spoilage and human fungal pathogen.

## Introduction

Biosurfactants are amphiphilic molecules comprised of a hydrophobic moiety (hydrocarbon- fatty acid with variable chain length) and variable hydrophilic moieties (phospholipid- phosphate group; neutral lipid- alcohol or ester group; fatty acid or amino acid- carboxylate group) (Ibacache-Quiroga et al., 2013). The biosynthetic gene cluster responsible for the synthesis and assembly of hydrophobic and hydrophilic moieties of biosurfactants varies depending on the microorganisms such as yeasts, bacteria, and fungi; they can produce a variety of biomolecules such as glycolipids, lipoproteins, lipopeptides, phospholipids, lipopolysaccharides (Ibacache-Quiroga et al., 2013; Van Bogaert et al., 2013). The glycolipids are the most studied biosurfactant and the best known are rhamnolipid, trehalolipid, mannosylerythritol lipids (MELs) and sophorolipids (SLs). SLs are mainly produced by non-pathogenic yeasts, e.g., *Candida batistae, C. apicola*, *Starmerella bombicola*, *Rhodotorula bogoriensis*, *Wickerhamiella domercqiae*, and *Rhodotorula babjevae* while MELs are produced by *Pseudozyma rugulosa*, *P. aphidis*, and *P. antacrtica* (Sen et al., 2017; Hipólito et al., 2020). SLs have wider commercialization value because of its higher production as compared to other glycolipid biosurfactants. They are comprised of a disaccharide sophorose associated to a terminal or sub-terminal fatty acid (chain length of usually 16–18 carbons) and are produced in lactonic (neutral) and acidic (anionic under alkaline conditions) forms (Hipólito et al., 2020; Twigg et al., 2020).

SLs have been recorded for a wide range of antimicrobial activities against various bacterial and fungal pathogens via., increased permeabilization, and membrane destabilization (Bluth et al., 2006). Earlier studies concerned with antifungal activities of SLs are somehow limited; therefore, there is necessity for more research to investigate the yeast species having high productiveness of biosurfactants and their applicability as an antifungal agent. The principal fungal pathogens such as *Fusarium*, *Colletotrichum*, *Aspergillus*, and *Botrytis* result in significant loss of wholesome stored fruits, grains and vegetables. The *Fusarium* genus causes the yellow and orange sporulation majorly in fruits and contaminates corn and roots (Hipólito et al., 2020). *Colletotrichum* is a major post-harvest pathogen which causes a significant loss of fruits such as mango, papaya, avocado, citrus and apple. It causes the brown rot on the infected areas initially, then the whole fruit may rot, leading to the development of fruiting bodies on the rotten surface (Tian et al., 2011). The continued use of agrochemicals has raised a serious concern for environment and food safety, so the plausible management strategies need to be investigated.

In this study, the yeast was isolated from insect gut because insect gut harbors an astonishing diversity of undescribed yeast species and is still grossly under-reported (Suh et al., 2005; De Vega et al., 2012). *Metschnikowia* clade is strongly associated with the insect-flower ecosystem (Gimenez jurado et al., 2003) and regularly encountered with the fruits, flowers, plant tissues, digestive tract, and frass of insects (Lachance, 2011). The yeast associated with the bees has a putative role as a producer of antimycotic substances which protect the bee hive from diseases (Hayden et al., 1997). So, analyzing the insect gut for novel yeast and their product might lead to more useful bioactive compounds. In the current study, a total of 3 strains were isolated from the gut of stingless bee, and they all represent one novel species. The biosurfactant of the strain CIG-6A^T^ was checked for the antifungal and antibacterial activity. To the best of our knowledge, this is the first report of antibacterial and antifungal activity of biosurfactant from *Metschnikowia* genus.

## Materials and Methods

### Isolation, Characterization and Identification of the Strain CIG-6A^T^

#### Isolation of Yeast Strains

The insect sample was collected from Churdhar, Himachal Pradesh, India, in 2016 and abbreviated as CIG^1^. The insects were placed in a sterile falcon without food for 2–3 days before dissection. The gut inhabiting yeasts were isolated using a previously described method (Suh and Blackwell, 2004). The homogenized sample was plated on yeast malt agar (YM), yeast peptone dextrose agar (YPD), and potato dextrose agar (PDA) supplemented with 100 mg/L of chloramphenicol to reduce the bacterial growth. The yeast colonies were purified, maintained, and preserved in 15% glycerol at −80°C and lyophilized for long-term storage (Saluja and Prasad, 2008).

#### Morphological and Physiological Characterization

The standard methods explained by Kurtzman were used to characterize the novel isolate (Kurtzman et al., 2011). Carbon assimilation tests were performed in Biolog YT microplate (Biolog, Inc., Hayward, CA) as per manufacturer instructions. Carbon fermentation and nitrogen assimilation tests were performed in a test tube using yeast nitrogen base (YNB), yeast carbon base (YCB) and starved inoculum. Sporulation was checked on different culture media such as YM agar, PDA, potato carrot agar, YCB with 0.01% ammonium sulfate, and cornmeal agar at 25°C for 21 days. The vegetative cell morphology and hyphae formation were observed using a confocal microscope (Nikon Instruments Inc., United States).

#### DNA Sequencing and Sequence Analysis

DNA isolation, PCR amplification, gel extraction, and sequencing of the ITS/D1D2 domain was performed using the method explained in Saluja et al. (Saluja and Prasad, 2008). The sequences obtained were submitted in the GenBank database and received the accession number MW244067 (ITS region) and MG821162 (D1/D2 region). The sequence of ITS/D1D2 region of the strain CIG-6A^T^ was compared with the GenBank database using the nBLAST^2^ and the MycoBank database using pairwise sequence alignment. Sequences of the closely related species of strain CIG-6A^T^ were retrieved from the GenBank and aligned by using CLUSTAL W (Thanh et al., 2002). The tree was constructed using Kimura two-parameter correction with 1,000 bootstrap values using the neighbor-joining method in MEGA Version 7.0 (Thanh et al., 2002; De Vega et al., 2012).

### Purification and Characterization of Biosurfactant CIG-6A^T^

#### Extraction and Purification

For the biosurfactant extraction, the culture grown for 72 h at 180 rpm at 25°C, was centrifuged (8,000 rpm for 30 min). Extraction was performed using cell-free supernatant with an equal amount of ethyl acetate (1:1 ratio) in the separating funnel. The organic layer was then separated and vacuum dried in a rotary evaporator at 45°C (Fontes et al., 2012). Biosurfactant was purified by silica gel (60–120 mesh) column chromatography (Daverey and Pakshirajan, 2010). Glass column with dimensions 45 × 3.5 cm^2^ was packed with silica gel in absolute methanol. The 5 ml of crude sample of biosurfactant (1 g), dissolved in methanol was loaded on the column and eluted by gradient system of methanol: chloroform (0–90% chloroform). The fractions were collected separately and vacuum dried at 45°C.

#### Biosurfactant Analysis by TLC (Thin Layer Chromatography)

The biosurfactant purified from silica gel (60–120 mesh) was dissolved in 100% methanol. The sample was spotted on the silica gel plate (Merck DC, Silica gel 60) and mobile phase used was chloroform: methanol: water (65: 25: 4, v/v). The silica plate was developed for lipid detection with fumes of iodine in a chamber, and later anthrone reagent was sprayed to detect the sugars. 1,4″-sophorolactone 6′,6″-diacetate (Sigma-Aldrich, United States) commercially available sophorolipid (SL-S) was used as a reference standard (Sen et al., 2017).

#### Fourier Transform Infrared Spectroscopy (FTIR)

The functional groups of biosurfactant were evaluated using infrared spectroscopy (FTIR system, Perkin Elmer, Branford, CT, United States). FTIR of standard SL-S along with the test biosurfactant in ATR (Attenuated total reflectance) was accomplished at a wavenumber and a resolution accuracy of 0.01 and 4 cm^–1^, respectively, and 32 scans with the association for atmospheric CO_2_ (Camargo et al., 2018). All of the data were corrected for the background spectrum.

#### Fatty Acid Analysis by GC-FID-MS (Gas Chromatography and Mass Spectra)

The esterified sample was used to determine the fatty acid composition of biosurfactant. The sample was prepared using the method explained in literature (Ribeiro et al., 2020). The sample was analyzed in gas chromatography with a flame ionization detector (GC/FID; Thermo-scientific TRACE 1300). The gas chromatograph equipped with DB-5 ms capillary column (30 m in length × 250 μm diameter × 0.25 μm) was used for analysis. The carrier gas used was helium at a flow rate of 1 ml/min. The detector and injector temperature were 320°C. The temperature of the oven was increased to 150°C at 10°C/min, and hold for 4 min, increased to 280°C at 4°C/min and held for 5 min. The fatty acids were identified using a standard external MIDI No. 1300-C mix C_9:0_–C_20:0_.

#### Liquid Chromatography-Mass Spectrometry (LC-MS)

Biosurfactant separation and structural homologs identification was done by LC-MS (1260 Infinity HPLC, Agilent Technologies, United States) using a reverse-phase, C18 column (250 mm × 10 mm, 150 Å, Waters) (Nuñez et al., 2004) along with standard SL-S. The mobile phase contained solvent A (water supplemented with 1% TFA) and solvent B (acetonitrile having 1% TFA). The gradient elution, i.e., 5–20% solvent B in 5 min, 20–80% B in 25 min, and reverse 80–5% B in 3 min was used in HPLC. Mobile phase flow rate was maintained at 3.0 ml/min. ESI-MS (Electrospray ionization- mass spectroscopy) was accomplished in positive ion mode, and spectral range from 200 to 800 m/z was examined through Agilent Mass Hunter software.

#### Ionic Character Determination of Biosurfactant

Agar double diffusion method was used to determine the ionic charge of biosurfactant (Santos et al., 2013). Two regularly spaced rows of well were made on agar plate with low degree of hardness (1%). The one row of wells was filled with biosurfactant and one row of wells was filled with compound of known ionic charge. The 20 mM SDS (Sodium dodecyl sulfate) was used as anionic compound and 50 mM barium chloride was used as cationic compound. The biosurfactant from the strain CIG-6A^T^ was dissolved in methanol; therefore, control of methanol was maintained under similar conditions with SDS and barium chloride.

### Physicochemical Properties of Biosurfactant CIG-6A^T^

#### Surface Tension (ST) Measurement

ST of biosurfactant was determined using tensiometer through Wilhelmy plate method at 25°C upto 192 h. For precise measurements, ultrapure water having ST of 72.8 mN/m was used to calibrate the instrument.

#### Critical Micelle Concentration (CMC)

The CMC was estimated by measuring the ST of the biosurfactant until a constant value of ST was reached. The ST of column purified biosurfactant was measured from 1 to 12 mg/ml concentration up to a ST’s constant value. The concentration at which ST value became constant was considered as CMC.

#### Emulsification Index (%E_24_)

The emulsification index (%E_24_) was analyzed, as reported earlier (Elshafie et al., 2015). Equal concentration of biosurfactant (2 ml) was added with different hydrocarbons (olive oil, crude oil and mineral oil) and vortexed at maximum speed for 3 min and kept at room temperature for 192 h. The emulsions formed were differentiated with negative control, i.e., YM broth, and positive control, i.e., Tween-20.

#### Stability Studies

The biosurfactant stability was examined in reference to pH, salinity, and temperature. The temperature stability, pH and NaCl tolerance of crude biosurfactant were investigated by incubating at different temperatures (37, 40, 60, 80, 100, and 121°C for 15 min), different pH (2.0–12.0) and NaCl concentrations (2–15% w/v), respectively.

### Antioxidant, Antimicrobial and Hemolytic Activity of Biosurfactant CIG-6A^T^

#### Antioxidant Activity of Biosurfactant Strain CIG-6A^T^

Free radical scavenging method was used to evaluate the antioxidant activity by using stable radical 2,2-Diphenyl-1-Picrylhyrdazyl (DPPH). The experiment was performed using the method according to Ribeiro et al. (2020). A stock solution of 200 μM DPPH was prepared in methanol. Different concentrations (2.5, 5.0, 10.0, and 20.0 mg/ml) of biosurfactant (40 μl) was mixed with 250 μl of DPPH to determine the antioxidant activity. L-ascorbic acid was used as standard and used in same concentration as biosurfactant from CIG-6A^T^. After 30 min of incubation in dark, the absorbance was read at 517 nm. The experiment was carried out in triplicates and percentage of inhibition was calculated using the following equations I% = [(Abs_0_ –Abs_1_)/Abs_0_] × 100. Abs_0_ is the control absorbance and Abs_1_ is absorbance in the presence of biosurfactant.

#### Determination of Minimum Inhibitory Concentration (MIC)

Biosurfactant MICs were evaluated using the microtiter plate dilution assay. Bacterial test strains used are *Staphylococcus aureus* (MTCC 1430), *Klebsiella pneumoniae* (MTCC 618), *Vibrio cholerae* (MTCC 3904), *Listeria monocytogenes* (MTCC 839), *Pseudomonas aeruginosa* (MTCC 1934), *Bacillus subtilis* (MTCC 121), *Bacillus cereus* (MTCC 9490), *Salmonella enterica* (MTCC 3232), *Escherichia coli* (MTCC 1610), and *Micrococcus luteus* (MTCC 106). Whereas, biosurfactant antifungal activity was checked against plant and human pathogens viz. *F. solani* (MTCC 350), *F. oxysporum* (MTCC 9913), *Penicillium chrysogenum* (MTCC 160), *C. gloeosporioides* (MTCC 2190), *Alternaria alternata* (MTCC 10576), and *Botrytis cinerea* (MTCC 2349) using a technique of broth microdilution (Santos and Hamdan, 2005). After 48 h, absorbance of the plate was measured at 600 nm through plate reader (BMG Labtech, Germany), and MIC was recorded as the lowest concentration at which growth was not observed.

#### Hemolysis Assay

Hemolysis assay was performed using a blood sample collected from rabbit (New Zealand White) in a test tube containing EDTA. The blood sample was centrifuged for 5 min at 1,550 rpm and washed thrice with phosphate buffer saline (PBS) (Baindara et al., 2016). 1X-PBS was used to prepare erythrocyte suspension (10%) and incubated along with the increasing biosurfactant concentrations at 37°C for 24 h. The samples were then centrifuged, and cell-free supernatants were used to examine the erythrocyte lysis at a 405 nm wavelength. PBS and 1% Triton X-100 were taken as negative and positive controls, respectively.

#### Confocal Laser Scanning Microscopy

Cells of *F. solani* were grown in the liquid medium to late logarithmic phase and 1 ml culture was centrifuged for 3 min at 1,000 g, washed and incubated with biosurfactant for two different time intervals (48 and 72 h) at 25°C. After each time point, cells were washed and resuspended in PBS containing PI (10 μmol^–1^) (Sen et al., 2020). Samples were incubated for 15 min and washed with PBS. Cells were suspended in PBS, and treated cells were observed microscopically (excitation wavelength 460–490 nm, emission wavelength > 520 nm). Untreated cells served as control.

#### Statistical and Structural Analysis

Data was represented as arithmetic average of minimum of three replicates and error bars defined standard deviations. The evaluation was executed using ANOVA, followed through the Tukey test with 95% of confidence level. For the present study, the biosurfactant’s chemical structures were drawn by using MarvinSketch version 20.19^3^. The MarvinSketch is a Java-based software used for chemical drawing and editing of molecules in various file formats.

## Results

### Isolation, Identification and Characterization of Strain CIG-6A^T^

#### CIG-6A^T^ Delineation and Identification

**FIGURE 1:**
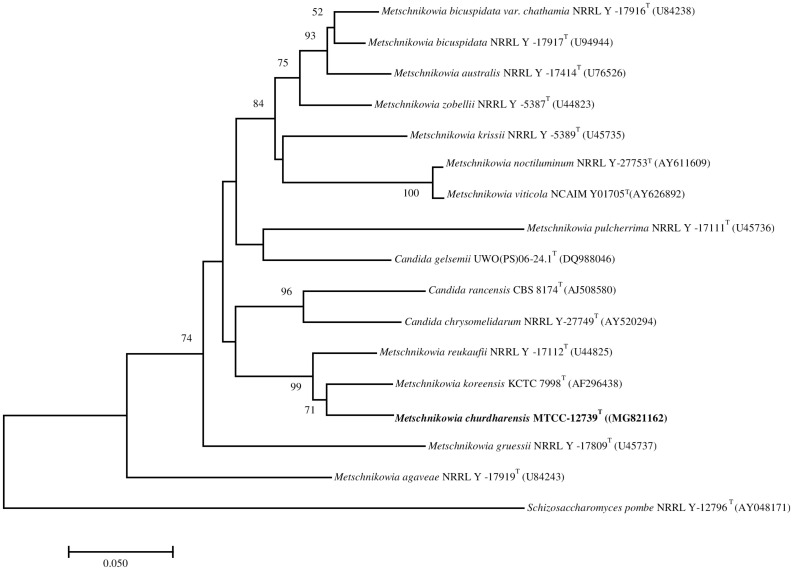
The phylogenetic tree was drawn using a neighbor-joining method based on sequences of D1/D2 domain of 26S rRNA gene, showing the relationship between *Metschnikowia churdharensis* f.a., sp. nov., strain CIG-6A^T^ and related members of the genus *Metschnikowia*. *Saccharomyces pombe* (T) **(AY041781)** was used as an out-group. Substitution per nucleotide position is bar 0.050. Bootstrap values (expressed as percentages of 1,000 replications) greater than 50% are given at nodes.

On pairwise sequence alignment of ITS region, strain CIG-6A^T^ showed 5.3% sequence divergence from *M. koreensis* (12 gaps and 3 substitutions) and 6.03% from *M. reukaufii* (7 gaps and 10 substitutions). The sequence of D1D2 region of the strain CIG-6A^T^ differed from *M. koreensis* by 5 to 5.5% (4 gaps and 24 substitutions) and 5.73% from *M. reukaufii* (8 gaps and 21 substitutions). The phylogenetic analysis based on the sequence of the D1D2 region of 26S rRNA gene placed the strain CIG-6A^T^ near to *M. koreensis* with 71% bootstrap support (Figure 1). Strain CIG-6A^T^ differs in nutritional requirements from the closely related species by the pattern of fermentation of cellobiose, maltose, sucrose, D-gluconic acid, D-galactose, and palatinose (Supplementary Table 1).

#### Description of *Metschnikowia churdharensis* f.a., sp. nov.

*Metschnikowia churdharensis* f.a., sp. nov., (chur.dhar.en’sis. N.L. fem. adj. *churdharensis*, related to the place from where it was isolated).

Colonies of the strain CIG-6A^T^ are raised, dull, smooth, cream colored, entire, and butyrous after 5 days of growth on YMA plate at 25°C (Supplementary Figure 1A). The cells are ovoid and occurred in short-chains or singly; budding is polar and measured 8–9 by 5–6 μm (Supplementary Figure 1B). On the cornmeal agar plate, after the incubation of 21 days at 25°C, pseudohyphae are produced.

D-glucose, D-cellobiose, maltose, sucrose, and D-mannitol are fermented. D-cellobiose, gentiobiose, maltose, maltotriose, β-methyl-D-glucoside, arbutin (weak), D-sorbitol, sucrose, melezitose (weak), D-glucose, N-acetyl-D-glucosamine (weak), D-glucosamine (weak), salicin (weak), and D-mannitol are assimilated. L-malic acid, L-glutamic acid, D-trehalose, α-keto-glutaric acid, L-arabinose, 2-keto-D-gluconic acid, inulin, D-galactose, xylitol, L-sorbose, melibiose, D-ribose, D-xylose, D-arabinose, erythritol, L-rhamnose, D-raffinose, and glycerol are not assimilated. Ethylamine, ammonium sulfate, cadaverine, nitrate (weak), lysine, nitrite (weak), creatinine (weak), and creatine (weak) are assimilated, and D-glucosamine and imidazole are not assimilated. In a vitamin free base medium, the growth is positive. Growth at 4, 12, 25, and 30°C is positive; at 37°C is negative. The acid production from glucose on custer’s chalk medium is negative (no clearing of the medium around the streak). On 10% NaCl agar and 16% NaCl agar, no growth is observed, and on 10% NaCl/5% glucose, the growth is positive or weak; with 16% NaCl/5% glucose, no growth is observed. The growth on 50% glucose is positive; with 60% glucose is positive or weak or delayed. Growth in 1% acetic acid medium is negative. Starch like compound formation is negative, and gelatin liquefaction is positive. Growth in 0.01 and 0.1% cycloheximide is negative. Diazonium blue B (DBB) reaction and urea hydrolysis are negative.

CBS 15318 is the holotype of *Metschnikowia churdharensis* f.a., sp. nov., and isotype is MTCC 12739. This strain was deposited in an inactive metabolic state in Microbial Type Culture Collection and Gene Bank (MTCC) Chandigarh, India and Westerdijk Fungal Biodiversity Institute, Utrecht, The Netherlands. The MycoBank number of the species is MB 824669.

### Physicochemical Properties of Biosurfactant CIG-6A^T^

**FIGURE 2:**
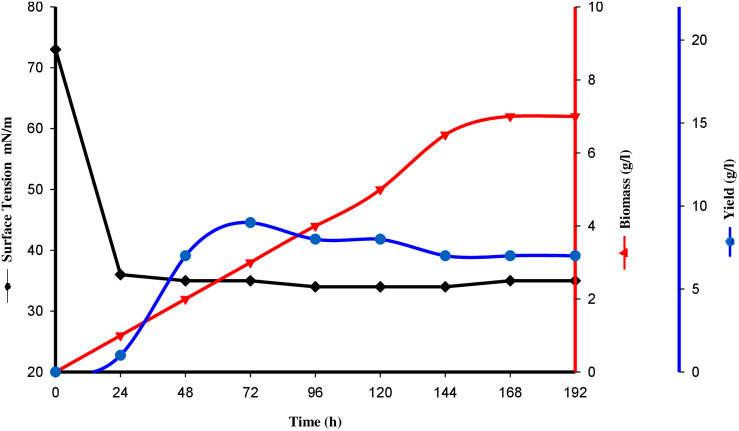
Surface tension, growth kinetics, biomass and yield of biosurfactant from the strain CIG-6A^T^ grown at 25°C, 180 rpm, 2% inoculum (v/v) plotted as a time function.

**FIGURE 3:**
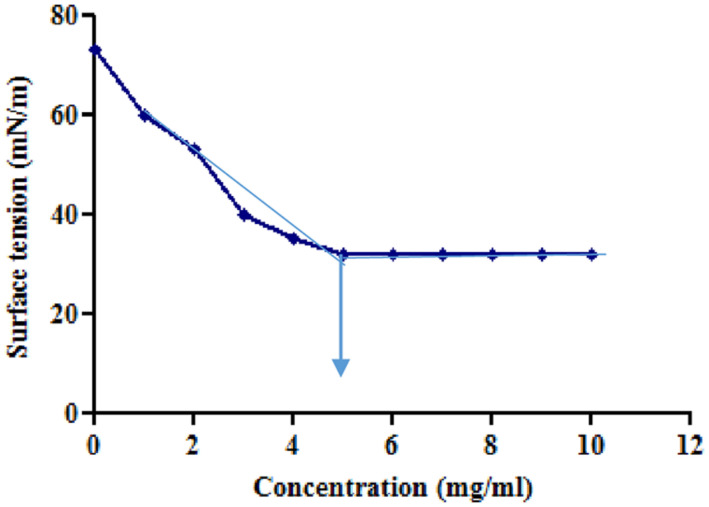
Minimum surface tension and CMC of the biosurfactant was evaluated. Arrow illustrate CMC value of crude biosurfactant produced by the strain CIG-6A^T^.

Tensioactive properties of the biosurfactant mainly relate to their potential to lower ST and CMC value. Biosurfactant from strain CIG-6A^T^ effectively reduced the ST from 72.8 to 35 mN/m. Growth kinetics demonstrated surface tension, biomass, and yield of biosurfactant of strain CIG-6A^T^ in Figure 2. As shown in Figure 3, CMC value was observed at 5 mg/ml, i.e., ST reduction was not observed any further on increasing the concentration of biosurfactant. Emulsification assay and emulsion stability after 24, 72, and 192 h were studied, as shown in Table 1. Biosurfactant was successfully stabilized and emulsified the formed emulsion with olive oil, mineral oil, and crude oil. E_24_ was highest against crude oil with 79% emulsion stability, whereas with mineral oil and olive oil, it was 60% emulsion stability. E_24_ was 0% with the negative control, i.e., media, and 100% with the positive control, i.e., Tween-20. The biosurfactant stability in terms of surface tension was evaluated. Thermal stability results revealed that biosurfactant surface tension was maintained up to 121°C (Supplementary Figure 2A). No change in surface tension was observed at pH 2.0, 4.0, 12.0, and 14.0 (Supplementary Figure 2B) and decreased surface tension was observed at pH 6.0, 8.0 and 10.0. No change in surface tension was observed till 10% of NaCl concentration (Supplementary Figure 2C).

**TABLE 1:** Emulsification index (EI) evaluated using biosurfactant produced form *Metschnikowia churdharensis* f.a., sp. nov., CIG-6A^T^ grown at 25°C, 180 rpm, 2% inoculum (v/v), 1% glucose (w/v), 1%NaCl (w/v) after 24, 72, and 192 h.

S.no	Hydrophobic substrates	Emulsification index (%)		
		24 h	72 h	192 h
1	Mineral oil	63 ± 0.11	63 ± 0.3	60 ± 0.12
2	Olive oil	65 ± 0.32	65 ± 0.2	63 ± 0.13
3	Crude oil	79 ± 0.21	77 ± 0.32	74 ± 0.22

### Characterization of Biosurfactant CIG-6A^T^

#### Thin-Layer Chromatography (TLC)

TLC was used as a prior methodology for biosurfactant compositional analysis. Lipids and sugars were detected by using iodine vapors and anthrone reagent, respectively. TLC chromatogram revealed the biosurfactant chemical form (Supplementary Figure 3) when compared with sophorolipid standard 1,4″-sophorolactone 6′,6″-diacetate (SL-S). In SL-S, two spots with R*_f_* values 0.657 and 0.710 were observed. In CIG-6A^T^ sample, spots were observed with R*_f_* values of 0.751 and 0.789, indicating lactonic SL’s presence, whereas spots appeared with R*_f_* value 0.105 and 0.167 indicating acidic SL in biosurfactant.

#### FTIR

**FIGURE 4:**
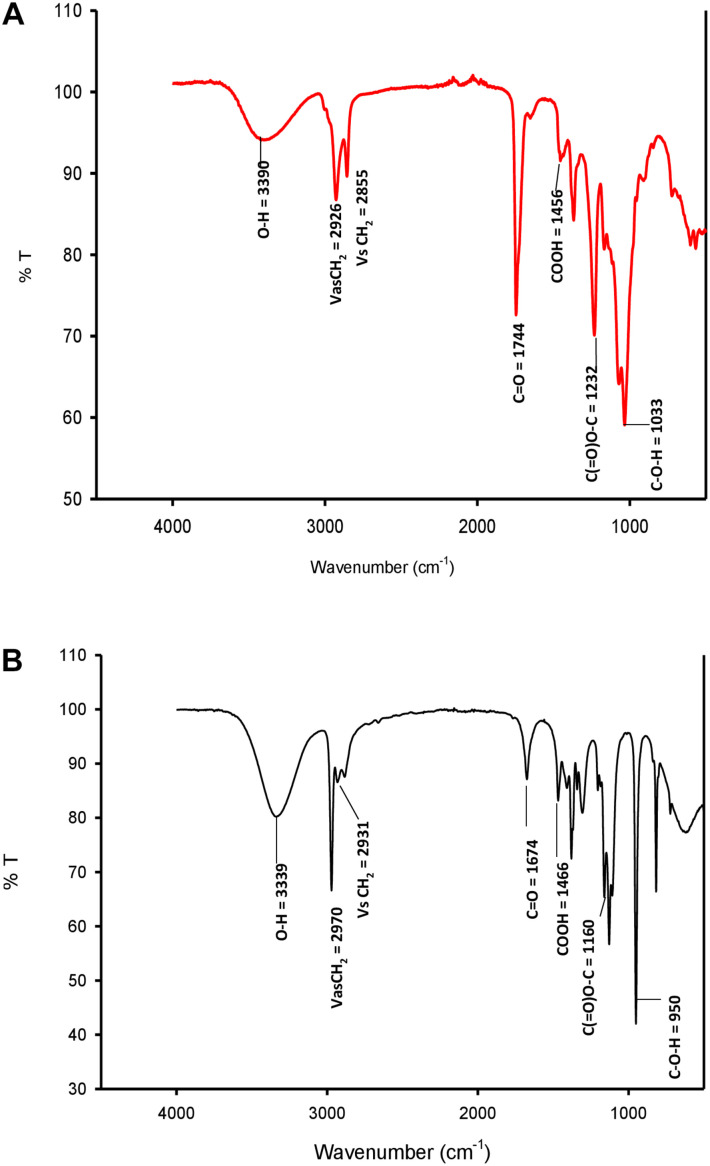
FTIR spectra of standard sophorolipid, 1,4″-sophorolactone 6′,6″-diacetate **(A)** and biosurfactant produced by the strain CIG-6A^T^ **(B)**.

FTIR spectra of SL-S (Figure 4A) and CIG-6A^T^ (Figure 4B). The absorption at 2,970 and 2931.65 cm^–1^ corresponds to symmetrical stretching (ν_s_ CH_2_) and asymmetrical stretching (ν_as_ CH_2_) of the methylene group, respectively. The absorption observed at 1674.35 cm^–1^ appears to be C = O lactone group. O-H stretch was represented by strong absorption at 3339.7 cm^–1^ as compared to the standard SL-S. C(= O)-O-C = stretch in lactone was observed by absorption at 1,160 cm^–1^. The absorption at 1466.8 cm^–1^ and 3339.7 reflect C-O-H stretch in-plane bending of carboxylic acid, might be typical acidic sophorolipid of CIG-6A^T^. Biosurfactant from CIG-6A^T^ was different from the standard in absorptions at 1674.35 and 950.66 cm^–1^ (C = O and C-O-H), respectively. FTIR data confirms that the biosurfactant from CIG-6A^T^ is both mixtures of acidic and lactonic SLs.

#### GC-FID-MS

**FIGURE 5:**
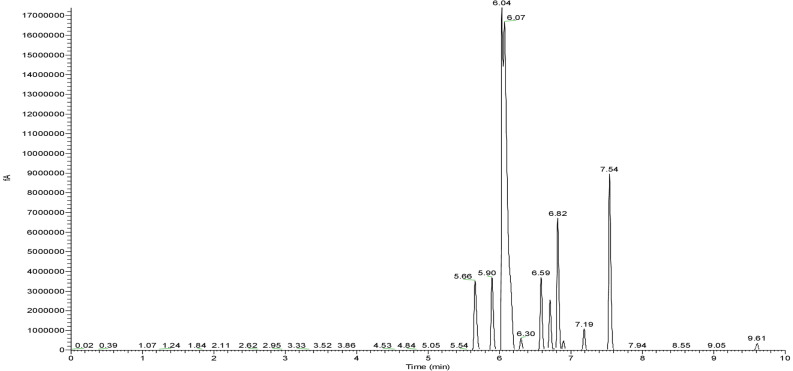
GC-FID-MS spectra of the biosurfactant produced by the strain CIG-6A^T^.

GC-MS spectra revealed the composition of fatty acids in biosurfactant (Figure 5). Total of eight fatty acids were found in CIG-6A^T^ biosurfactant. The palmitic acid C_16:0_ and linolenic acid C_18:3_ were the predominant fatty acids in biosurfactant, whereas C_10:1_, C_10:2_, C_13:0_, C_15:1_, C_15:3_, and C_18:2_ fatty acids were present in lesser proportion.

#### LC-MS

**FIGURE 6:**
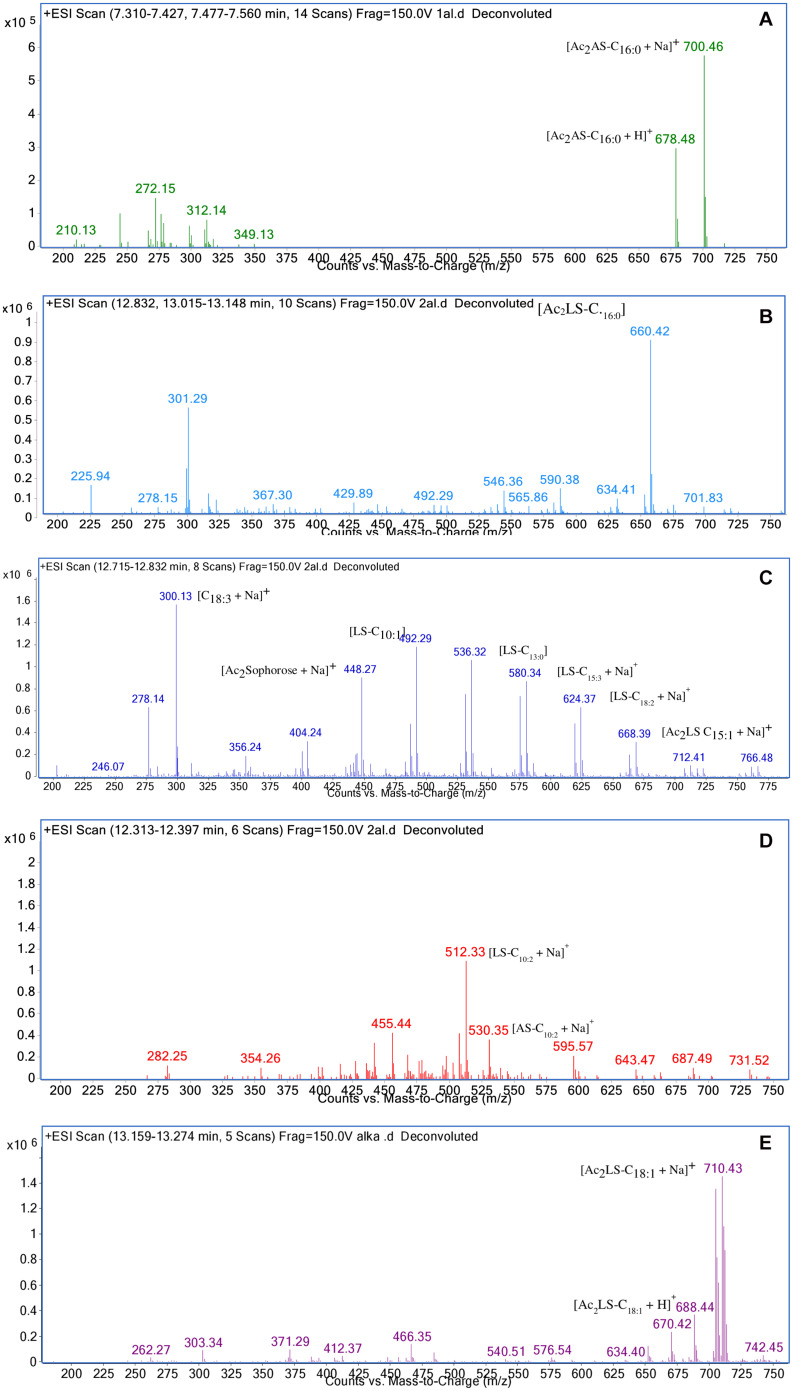
Characterization of the biosurfactant produced by the strain CIG-6A^T^ using LC-MS in positive electrospray ionization mode (+ ESI). **(A)** MS showing the sodiated adducts of diacetylated acidic sophorolipids (AS) with hexadecanoic acid (C_16:0_) lipid side chain at m/z values 678 and 700. **(B)** Diacetylated lactonic form of hexadecanoic acid (C_16:0_) chain was detected at m/z 660. **(C)** The ion at m/z 492 and 536 corresponds to lactonic sophorolipid with decanoic acid and tridecanoic acid, respectively with lipid chain (LS-C_10:1_ and LS-C_13:0_). Two sodiated adducts of lactonic sophorolipid with pentadecanoic acid and octadecanoic acid, respectively with lipid chain (LS-C_15:3_ and LS-C_18:2_) were detected. Diacetylated sodiated adduct of sophorose moiety was observed at m/z 448 **(D)** Sodiated sophorolipid with the acidic and lactonic form with a decanoic fatty acid side chain (AS-C_10:2_) was observed at m/z 530 and 512, respectively. **(E)** LC-MS spectra of the standard sophorolactone (SL-S), 1,4″-sophorolactone 6′,6″-diacetate in positive electrospray ionization mode (+ ESI). MS showing the protonated ion and sodiated adducts of di-acetylated lactonic sophorolipids (Ac_2_LS) with octadecenoic (C_18:1_) lipid side chains at m/z values 688 and 710, respectively.

**FIGURE 7:**
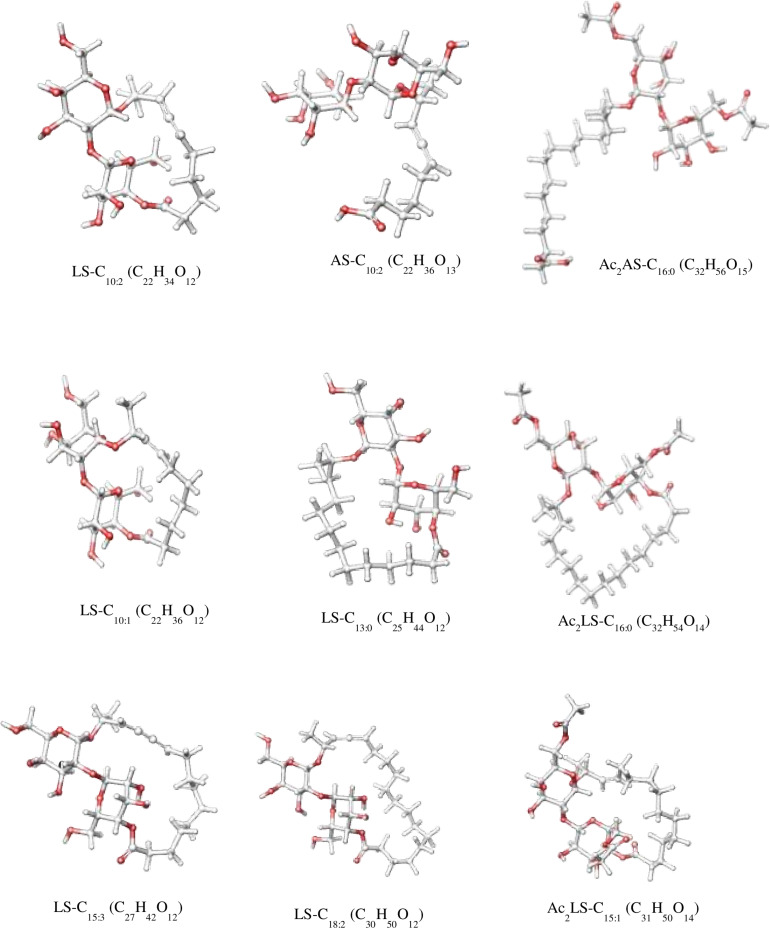
The least energy structures of the sophorolipid homologs detected during LC–MS analysis of the SL produced by the strain CIG-6A^T^. Structures were drawn in Marvin sketch 20.19 (*LS* lactonic SL, *AS* acidic SL, *Ac* acetyl group).

In sample CIG-6A^T^ as well as SL-S, LC-ESI-MS were acquired in positive mode. It revealed the presence of protonated and sodiated adduct ions in both sample and the standard. MS of CIG-6A^T^ biosurfactant was analyzed at different retention time points (Figure 6). Purified biosurfactant was a mixture of nine components. The sample contained seven lactonic and two acidic sophorolipid having a lipid chain of variable unsaturation and lengths. At the retention time (7.51 min), a diacetylated sodiated form of acidic sophorolipid was observed. Peak at m/z 678 and m/z 700 appears to [M + H]^+^ ion of diacetylated acidic sophorolipid Ac_2_AS C_16:0_ + H^+^ and [M + Na]^+^ ion of Ac_2_AS C_16:0_ + Na^+^, respectively as shown in Figure 6A. At the same retention time, peaks at m/z 210, 272, and 312 correspond to tridecanoic, octadecanoic and eicosanoic fatty acid chain fragments, respectively. Additionally, diacetylated lactonic form of hexadecanoic acid (C_16:0_) lipid chain was detected at m/z 660 (Figure 6B). The peak at retention time 12.7 min corresponds to adducts of lactonic sophorolipids with variable length of unsaturated fatty acids viz., m/z 448.2, 492.29, 536.32, 580.34, 624.37, and 668.39 are equivalent to Ac_2_ sophorose + Na^+^, LS C_10:1_, LS C_13:0_, LS C_15:3_ + Na^+^, LS C_18:2_ + Na^+^, and Ac_2_ LS C_15:1_ + Na^+^, respectively (Figure 6C). Fatty acids with m/z 300.13 correspond to C_18:3_ + Na^+^ and m/z at 278.14 equivalent to C_18:3_ detected at the same retention time. Retention time 12.2 min represents the lactonic and acidic form of CIG-6A^T^ sophorolipid with chain length LS-C_10:2_ and AS C_10:2_ with 18 Dalton difference at m/z 512 and 530, respectively (Figure 6D). Sophorolipids from SL-S were detected under the same conditions as showed in Figure 6E. The peaks at m/z 710.4 and 688 with RT 13.1 min correspond to [M + Na]^+^ and [M + H]^+^ ions of Ac_2_ sophorolactone with C_18_ saturated fatty acid moiety. The finalized sophorolipid homologs list detected in CIG-6A^T^ with their least energy structures are shown in Figure 7 and Table 2.

**TABLE 2:** Type of sophorolipid homologs, Molecular mass and chemical structure of sophorolipid produced by the strain CIG-6A^T^, along with sophorolipid standard, 1,4″-sophorolactone 6′,6″-diacetate (SL-S) as determined by LC-MS.

Homolog	Formula	Molecular mass	Type of SL	Source
AS-C_10:2_	C_22_H_36_O_13_	508.52	Acidic	SL-CIG-6A
LS-C_10:2_	C_22_H_34_O_12_	490.5	Lactonic	SL-CIG-6A
Ac_2_LS-C_16:0_	C_32_H_54_O_14_	660.8	Lactonic	SL-CIG-6A
Ac_2_AS-C_16:0_	C_32_H_56_O_15_	678.8	Acidic	SL-CIG-6A
LS-C_10:1_	C_22_H_36_O_12_	492.52	Lactonic	SL-CIG-6A
LS-C_13:0_	C_25_H_44_O_12_	536.62	Lactonic	SL-CIG-6A
LS-C_15:3_	C_27_H_42_O_12_	558.62	Lactonic	SL-CIG-6A
LS-C_18:2_	C_30_H_50_O_12_	602.72	Lactonic	SL-CIG-6A
Ac_2_LS-C_15:1_	C_31_H_50_O_14_	646.73	Lactonic	SL-CIG-6A
Ac_2_LS-C_18:1_	C_34_H_56_O_14_	688.8	Lactonic	SL-S

**LS lactonic sophorolipid, AS acidic sophorolipid, Ac acetyl group. SL-CIG-6A, sophorolipid produced by Metschnikowia churdharensis f.a., sp. nov., SL-S, sophorolipid standard.**

#### Ionic Character of Biosurfactant CIG-6A^T^

Double diffusion agar test revealed the appearance of precipitation line between selected anionic compound (SDS) and the biosurfactant produced by the strain CIG-6A^T^. Simultaneously, no line was observed between the cationic compound (barium chloride) and the biosurfactant from strain CIG-6A^T^.

### Antioxidant, Antimicrobial and Hemolytic Activity of Biosurfactant CIG-6A^T^

#### Antioxidant Activity

**FIGURE 8:**
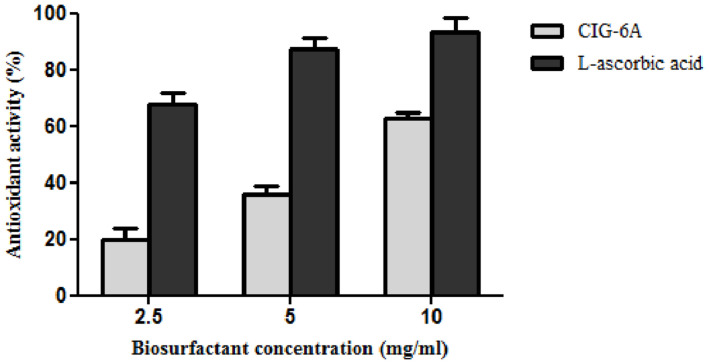
Antioxidant activity of biosurfactant sophorolipid from strain CIG-6A^T^ in comparison with L-ascorbic acid using DPPH assay.

DPPH method was used to evaluate the antioxidant property of biosurfactant using L-ascorbic acid as a standard and DPPH as control (Figure 8). The biosurfactant from CIG-6A^T^ showed maximum antioxidant activity of 62.98% (10 mg/ml) after 30 min of incubation, while the standard showed 93% of activity.

#### Antimicrobial and Hemolytic Activity of Strain CIG-6A^T^

**FIGURE 9:**
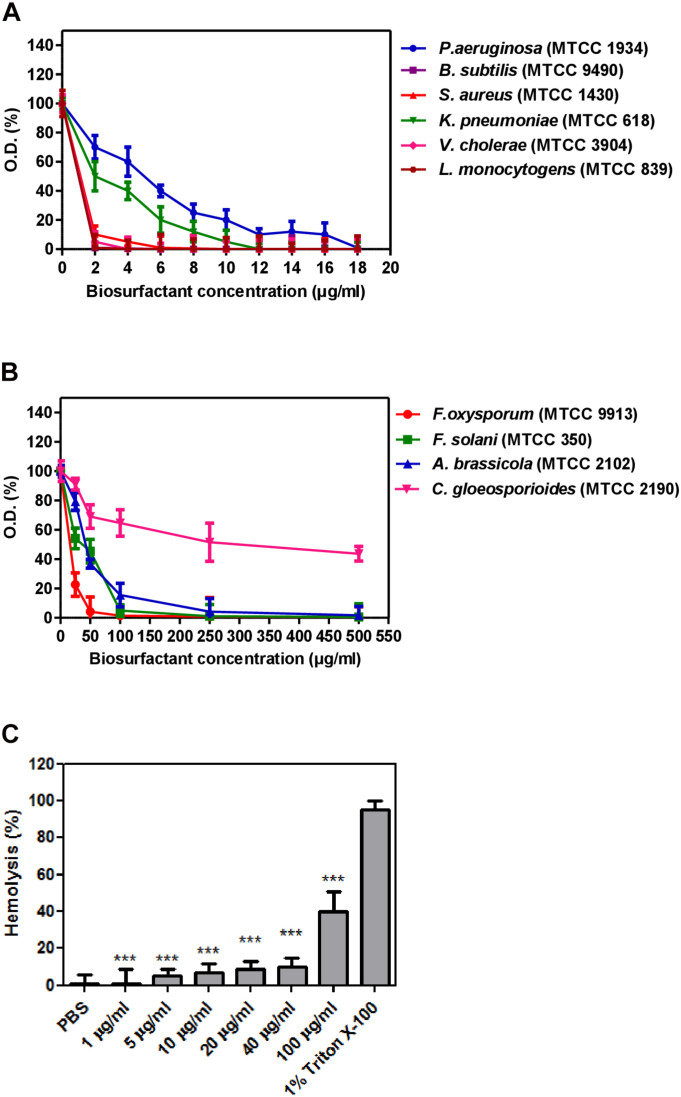
Determination of minimum inhibitory concentrations (MICs) and hemolysis activity of biosurfactant produced from the strain CIG-6A^T^ in a microtiter plate assay performed in triplicate. **(A)** MICs against Gram-negative and Gram-positive bacteria. **(B)** MICs against pathogenic fungal strains. **(C)** Hemolysis assay of biosurfactant using RBCs of a rabbit. Purified biosurfactant and RBC samples were prepared in PBS. All the experiments are executed in triplicate. Bars show SD. *P*-values are showed above each bar (significance was at a P level of 0.05). ***p ≤ 0.001.

Biosurfactant from strain CIG-6A^T^ displayed broad-range activity against Gram-negative and Gram-positive bacteria. The MIC analysis of biosurfactant showed that it is more effective against *S. aureus* and *B. subtilis* with complete inhibition at 5 and 1 μg/ml concentration, respectively, whereas 12 and 18 μg/ml concentration of biosurfactant were required to inhibit *K. pneumonia* and *P. aeruginosa*, respectively (Figure 9A). Biosurfactant of CIG-6A^T^ showed activity against human and plant fungal pathogens, evaluated on MIC basis (50–1,000 μg/ml). CIG-6A^T^ biosurfactant revealed optimistic antifungal activity against *F. oxysporum* and *F. solani* as observed from low MIC value (Figure 9B). Hemolysis assay results did not appear to have any lysis of RBCs (Red blood cells) as shown in Figure 9C even at high MICs values.

#### Confocal Laser Scanning Microscopy

**FIGURE 10:**
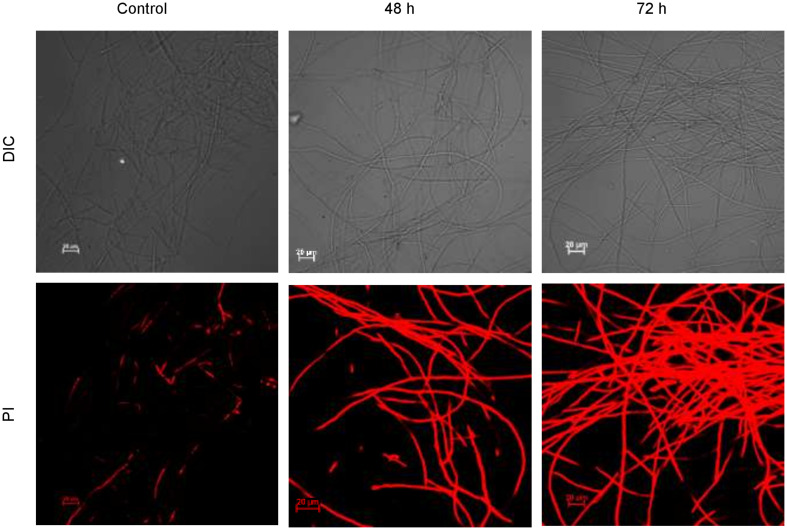
Confocal laser scanning microscopic (CLSM) images of the inhibitory effect of biosurfactant from the strain CIG-6A^T^ against *Fusarium solani*. Scale bar = 20 μm.

CLSM images (Figure 10) exhibited permeation of PI that increased the red fluorescence on the treatment with biosurfactant from strain CIG-6A^T^, after 48 and 72 h of incubation, indicating the death of the cells. Whereas, untreated samples revealed no red fluorescence, suggesting healthy cells.

## Discussion

The phylogenetic analysis and biochemical characteristics comparison revealed that the strain CIG-6A^T^, isolated from the gut of stingless bee, represents a novel yeast species. The insect samples were collected during the summer season from Churdhar (30° 52′ 34.68″ N, 77° 24′ 4.68″ E) Himachal Pradesh, India, belonging to a very cold and high-altitude place. The *Metschnikowia* clade is diverse in morphological, physiological characteristics, and rRNA gene sequence. This diversification of *Metschnikowia* clade is mainly due to the expansion of flowering plants and insects associated with them (Sipiczki, 2014; Álvarez-Pérez et al., 2015). Different *Metschnikowia* clade members, i.e., *M*. *pulcherrima*, *M*. *gruessii*, *M*. *reukaufii*, and *C*. *rancensis* were most commonly present in the gastrointestinal tract of bumblebees. Yeasts use this symbiosis to survive during winters when flowers and active insects are absent (Brysch-Herzberg, 2004). *Metschnikowia* species provide nutritional factors to the insects and suppress the growth of opportunistic microorganisms that may hamper their symbiosis (Brysch-Herzberg, 2004). There are 26 *Candida* and 39 *Metschnikowia* species in *Metschnikowia* clade (Lachance, 2011; De Vega et al., 2012). The estimated global number of insects in Hymenoptera order is around 1,15,000, and estimated species are approximately more than 3,00,000 in numbers (Chapman, 2009). The total number of bees species accepted are about 20,000, and most of them were never studied for the yeast’s presence (Rosa et al., 2003). Continued research on the yeast communities associated with bees might discover new species and their exploitation for bioactive compounds.

This study reports the novel yeast species, *Metschnikowia churdharensis* f.a., sp. nov., of strain CIG-6A^T^ having the ability to produce biosurfactant for the first time, which eventually characterized as sophorolipid with significant antifungal activity and having stable tensioactive properties. *Metschnikowia* clade has not been explored and characterized for the biosurfactant production, its antimicrobial and antifungal activity.

We analyzed physicochemical properties of biosurfactant from strain CIG-6A^T^ based on surface tension reducing ability, CMC, and emulsification activity. The potential biosurfactant is anticipated to reduce ST to nearly 35 mN/m (Camargo et al., 2018). Consequently, strain CIG-6A^T^ reduced ST below 35 mN/m value recommended a successful biosurfactant production. CMC equivalent to minimum surfactant concentration where monomers of surfactant begin forming micelles, at this point, medium solution interface in which biosurfactant diffused gets saturated with molecules of surfactant (Rufino et al., 2014). After CMC, no crucial ST lowering is observed, and low CMC value specifies a highly effective surfactant (Santos et al., 2018). CMC value acquired in our study was similar to biosurfactant produced by *Pediococcus dextrinicus* (Ghasemi et al., 2019). Emulsification is one of the properties of biosurfactant, which creates an emulsion of two immiscible liquids and increases bioavailability. Biosurfactant from strain CIG-6A^T^ contributed high%E_24_ with crude oil and low%E_24_ with mineral oil. Similar results were observed with biosurfactant produced from *Candida bombicola* (Elshafie et al., 2015).

Biosurfactant stability was assessed against a set of variable conditions, such as temperature, pH, and NaCl. The autoclaved sample had a minor change in surface tension. At the same time, a heated sample at 100°C has slight change in surface tension. Surface tension was stable at some pH and was relatively stable at all NaCl concentrations (as described above). Therefore, sophorolipid manifested magnificent stability over the evaluated range of temperature, pH and NaCl, suggested that there is a possibility of biosurfactant usage in food formulation.

Chemical characterization of biosurfactant produced by strain CIG-6A^T^ was performed using TLC, FTIR, GC-FID-MS, and LC-MS analysis regulated in contrast with SL-S. Sophorolipid produced by CIG-6A^T^ was a combination of lactonic and acidic sophorolipids analyzed through TLC. Sen et al. (2017) acquired R*f* values of acidic SL produced by *R. babjevae* in a similar range of 0.13–0.18, separated with the same mobile phase as used in this study.

The functional groups present in purified biosurfactant were estimated through FTIR and differentiated from the standard. Bands obtained in the sample and standard confirmed the lactonic SL presence. In contrast, acidic SL was confirmed through the presence of two bands at 3339.79 and 1466.8 cm^–1^, often associated with the acidic SL in the literature (Daverey and Pakshirajan, 2009). Purified biosurfactant was analyzed by gas chromatography for the presence of different fatty acids and the gas chromatogram showed biosurfactant comprised of palmitic acid (C_16:0_), capric acid (C_10:1_), and linolic acid (C_18:2_) and the predominant fatty acid present was palmitic acid (C_16:0_). On ESI of small molecules, having a single functional group able to carrying electrical charge involves proton addition to the analyte (M + H^+^) and cations adduction such as M + Na^+^ due to salt presence (Nuñez et al., 2004). Similarly, our study observed both protonated and sodiated ions of lactonic and acidic SLs with different side chains of fatty acids (C_10_–C_18_). Similar sophorolipid ions with heterogenous composition were also observed for *R. babjevae* YS3 through LC-MS analysis (Sen et al., 2017). A mixture of sophorolipids was obtained in a production medium containing both lactonic and acidic sophorolipids, but acidic sophorolipid represent the most considerable fraction of the product. In our study, LC-MS analysis also confirms a similar composition for sophorolactone standards as previously reported (Sen et al., 2017). Biosurfactants can be non-ionic, anionic, or cationic in their hydrophilic portions, while their hydrophobic part composed of a branched or linear chain of hydrocarbons (C8–C18 carbon atoms). Anionic biosurfactant is very common in the genus *Candida* of yeast (Camargo et al., 2018) whereas cationic biosurfactants are rare in nature. The ionic character determination revealed that the biosurfactant from CIG-6A^T^ is cationic and reported for the first time from *Metschnikowia* clade. Regarding the results of DPPH sequestration, biosurfactant CIG-6A^T^ presented the capacity to donate the hydrogen, therefore, showed DPPH scavenging activity. As compared to the natural antioxidant such as L-ascorbic acid, biosurfactant seems to be less effective but it can be used as a good alternative for synthetic antioxidants.

Many microorganisms producing biosurfactants have been explored for their antimicrobial properties and are presently exploited to stem the incidences of antibiotic resistance afflicted today’s world (Desai and Banat, 1997). Biosurfactants exhibited antagonistic activities that may be due to cellular membrane destabilization, which causes the extrusion of cytoplasm and, at last, results in rupturing of the cell (Hirata et al., 2009). Dengle-Pulate et al. (2014) reportedly used 1 and 6 μg/ml concentration to obtain complete *B. subtilis* and *S. aureus* inhibition, respectively and similar results were obtained in our study. There are very few studies on the antifungal activity of sophorolipids from yeast. We studied the antifungal activity against *F. oxysporum* and *F. solani*, which are the pathogens associated with post-harvest spoilage of vegetables like beans, potato and tomato (Kakde and Kakde, 2012). The promising antifungal activity was observed against plant and human pathogen *F. oxysporum* and *F. solani* at 49 and 98 μg/ml, respectively, indicates that it could have a possible application as a protective agent against post-harvest disease-causing fungal pathogens. The membrane integrity and viability can be determined by using PI, a membrane impermeable fluorescence dye, which binds to the nucleic acid, therefore differentiate damaged cells from healthy cells. Upon treatment with biosurfactant from CIG-6A^T^, live and dead cells of *F. solani*, stained with PI were visualized by CLSM, that confirm the irreversible damage to membrane integrity and structure damage. Previously, (Sen et al., 2020) demonstrated permeabilization of *Trichophyton mentagrophytes* membrane on treatment with sophorlipid produced by *R. babjevae*. Sophorolipid from CIG-6A^T^ has seven homologs of lactonic analog in contrast to two homologs of acidic analog, giving it more lipophilic character. It has been reported that lipophilic character of antifungal compound increased membrane permeability which hampers the ion transportation and latterly causing cell death (Di Pasqua et al., 2007). Therefore, biosurfactant sophorlipid from CIG-6A^T^ might exert its effect by changing the permeability of the fungal cells, thereby causing cell death.

This is the first study to demonstrate biosurfactant action against *F. oxysporum*, *F. solani*, *P. chrysogenum*, and *C. gloeosporioides* species capable of food spoilage and mycotoxin production. Speices of *fusarium* are capable of producing mycotoxins such as trichothecenes and fumonisins, which are often associated with toxicoses in humans and livestock. Trichothecenes causes toxicity to plants and animals by inhibiting ribosomal protein synthesis, RNA and DNA biosynthesis, and mitochondrial function. Fumonisins is a potential carcinogenic mycotoxin for human and causes lethal livestock diseases (Munkvold, 2017). The biosurfactant obtained from the present study appear to be more potent against food spoilage, plant and human fungal pathogens. Therefore, there is a new possibility for the application of biosurfactant as an alternative natural fungicide agent.

## Conclusion

In this study, *Metschnikowia churdharensis* f.a., sp. nov., CIG-6A^T^ was isolated from the gut of stingless bee (Hymenoptera order). The CIG-6A^T^ showed the production of sophorolipid biosurfactant, which was described as thermostable, emulsion stabilizing, and antioxidant agent, thereby have potential use in food industry. Moreover, sophorolipid biosurfactant showed promising results against food spoilage fungal pathogens. Based on the promising results obtained in the study, this sophorolipid biosurfactant has biotechnological potential for the food industry.

## Data Availability Statement

The datasets presented in this study can be found in online repositories. The names of the repository/repositories and accession number(s) can be found below: https://www.ncbi.nlm.nih.gov/genbank/, MG821162.

## Author Contributions

AK and SK conceived, designed, and performed the experiments. AP and GP supervised the project and provided the funding’s. AP, GP, AK, and SK analyzed the data and wrote the manuscript. All authors approved the submitted version of research article.

## Conflict of Interest

The authors declare that the research was conducted in the absence of any commercial or financial relationships that could be construed as a potential conflict of interest.

## Footnotes

1 CIG, Churdhar Insect Gut.

2 nBLAST, nucleotide Basic Local Alignment Sequencing Tool.

3 http://www.chemaxon.com/products/marvin/marvinsketch/
