# Supplementary material for: Production of Sophorolipid Biosurfactant by Insect Derived Novel Yeast Metschnikowia churdharensis f.a., sp. nov., and Its Antifungal Activity Against Plant and Human Pathogens
Source: Front Microbiol. 2021 Jun 4;12:678668. doi: 10.3389/fmicb.2021.678668 (PMC8212020; doi:10.3389/fmicb.2021.678668)
Supplement: Supplementary file 1 [file Data_Sheet_1.docx]

**Supplementary Material**

**Supplementary Figure 1A**: Colonies of strain CIG-6A^T^ grown on YM agar for three days at 25°C.


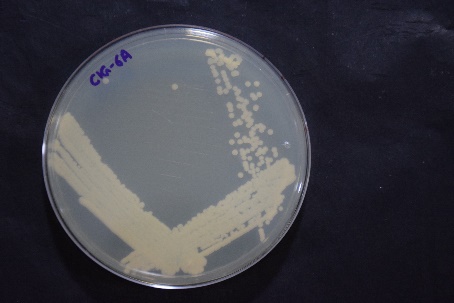


**Supplementary Figure 1B**: Micrograph of cells of *Metschnikowia churdharensis* f.a., sp. nov. The cells were grown on YM agar for two days at 25°C.

**
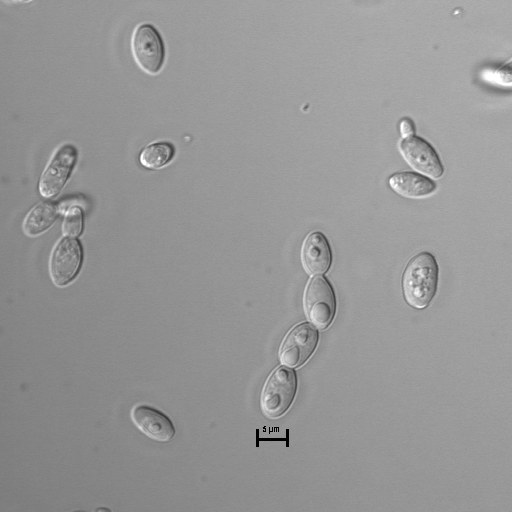
**

**Supplementary Figure 2:** Temperature (**A**), pH (**B**) and NaCl (**C**) Stability of biosurfactant activity produced by the strain CIG-6A^T^ against *K. pneumoniae.* Error bars demonstrate SEM (standard error of the mean), calculated from the two experiments performed in triplicates.

**(A)**

**(B)**

**(C)**

**Supplementary Figure 3:** Thin layer chromatography (TLC) exhibiting components separation of sophorolipid biosurfactant produced by *Mestchnikowia churdharensis* CIG-6A^T^ compared to standard of sphorolipid, viz., 1,4′′-sophorolactone 6′,6′′-diacetate.

CIG-6A SL


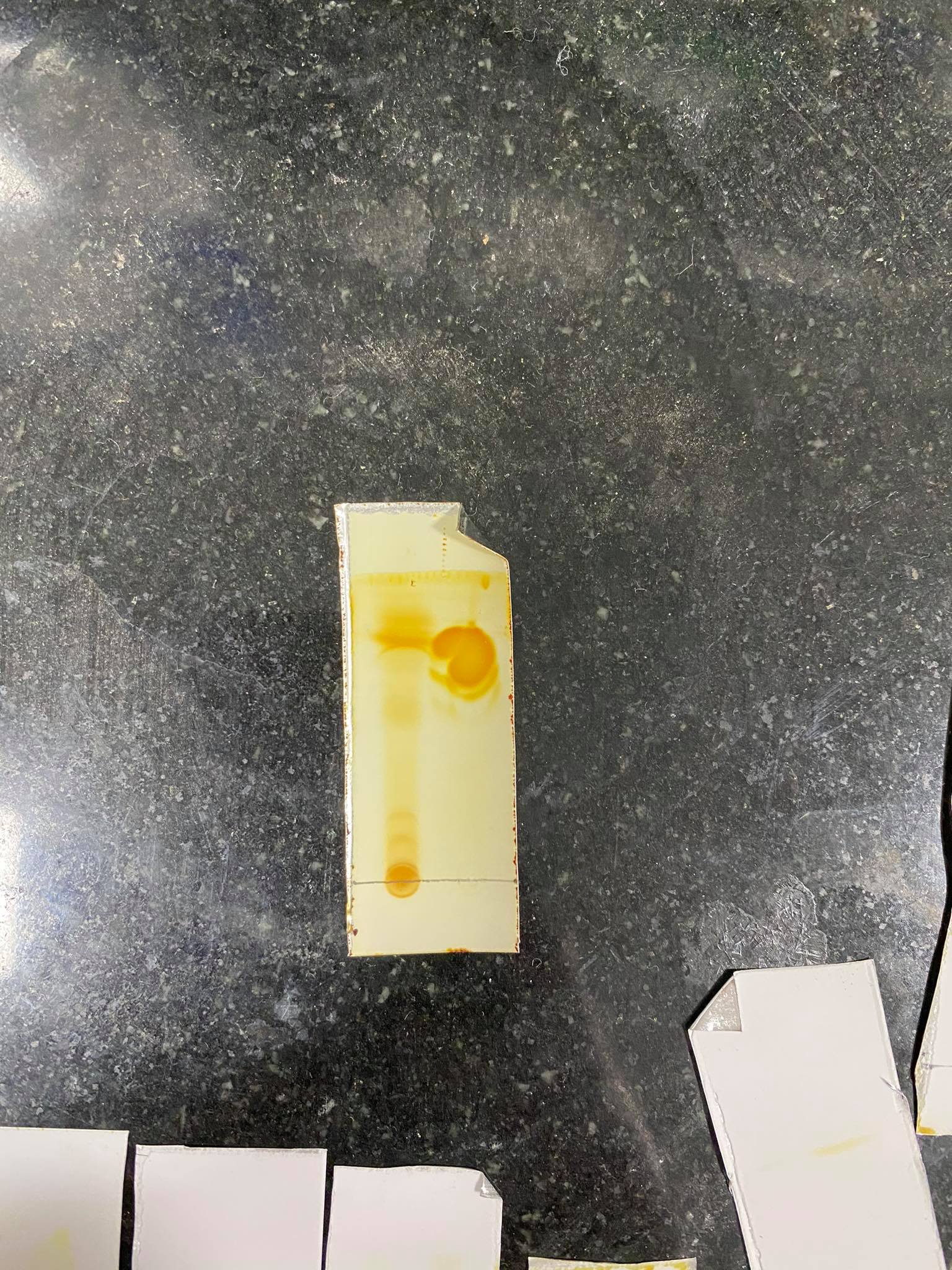


0.710

0.657

0.59

0.789

0.751

0.105

0.167

**Supplementary** **Table 1:** Biochemical characteristics of *Metschnikowia churdharensis* f.a., sp. nov., MTCC-12739^T^ and their closely related type strain.

w = weak; d = delayed. Strains *Metschnikowia churdharensis* f.a., sp. nov., MTCC-12739^T^, *Metschnikowia koreensis* CBS 8854^T^ (Hong *et al.* 2001)

| **Physiological test** | **MTCC-12739^T^** | **CBS 8854^T^** |
| --- | --- | --- |
| **Fermentation of carbon compounds** | | |
| Glucose | + | + |
| Maltose | + | - |
| Sucrose | + | - |
| **Assimilation of carbon compounds** | | |
| D- Galactose | - | + |
| D-Glucose | + | + |
| D-Glucosamine | - | + |
| D-Ribose | - | - |
| D-xylose | - | + |
| Maltose | + | + |
| D-Cellobiose | + | + |
| Melizitose | - | + |
| Salicin | - | + |
| Sucrose | + | + |
| Glycerol | - | + |
| **Assimilation of nitrogen compounds** | | |
| Nitrite | w/d | - |
| Nitrate | w/d | - |
| Cadaverine | + | + |
| D-Glucosamine | - | + |
| 25°C | + | + |
| 30°C | + | + |
| 35°C | - | + |
| 37°C | - | - |
| 10% Na cl | - | + |
| 50% Glucose | + | + |
| Acid production from glucose | - | - |
